# Constraint-Induced Movement Therapy Combined With Anodal Transcranial Direct Current Stimulation and Peripheral Neuromuscular Electrical Stimulation in Poststroke Patients: A Retrospective Study

**DOI:** 10.7759/cureus.79112

**Published:** 2025-02-16

**Authors:** Atsushi Umeji, Satoru Amano, Yukihisa Hashimoto, Yuki Uchiyama, Kazuhisa Domen

**Affiliations:** 1 Rehabilitation, Hyogo Medical University Hospital, Nishinomiya, JPN; 2 Rehabilitation, School of Allied Health Sciences, Kitasato University, Sagamihara, JPN; 3 Rehabilitation Medicine, Hyogo Medical University, Nishinomiya, JPN

**Keywords:** constraint induced movement therapy, rehabilitation, retrospective studies, storke, upper lim

## Abstract

Objectives: Although previous studies have shown a certain effect for affected upper extremity motor function for constraint-induced movement therapy (CIMT) with transcranial direct current stimulation (tDCS), there is insufficient evidence to make treatment recommendations. Here, we aimed to determine whether the addition of anodal-tDCS and peripheral neuromuscular electrical stimulation (PNES) to CIMT is superior to CIMT alone for improving upper extremity function in patients with chronic stroke.

Methods: This retrospective study included patients with chronic hemiparesis following a stroke who underwent CIMT at a college hospital between 2012 and 2018. The participants had either received CIMT alone (five-hour training sessions per day for 10 consecutive weekdays) or CIMT combined with anodal-tDCS and PNES.

Results: A total of 25 patients met all eligibility criteria, and 19 and 6 patients were included in the CIMT alone and CIMT combined with anodal-tDCS and PNES groups, respectively. Both groups showed significant improvement in all outcomes following CIMT (p < 0.05). The Fugl-Meyer assessment showed a significant difference between the groups in the CIMT combined with anodal-tDCS and PNES group (p = 0.047).

Conclusions: Preconditioning interventions, including tDCS and PNES, may be good methods for further enhancing the effectiveness of CIMT.

## Introduction

Stroke is recognized as a cause of various complications and long-term disability [1]. Motor impairments, including hemiparalysis, are the most common impairments caused by stroke. This can sometimes cause disability and impact activities of daily living (ADLs) [2], as well as quality of life [3]. Therefore, developing more effective treatments for motor impairments is a significant issue in rehabilitating patients following stroke.

Interhemispheric competition assumes that motor impairments may be related to both a reduced output from the damaged hemisphere and a disproportionate inhibition from the unaffected hemisphere [4]; therefore, modifying cortical activities for motor function recovery following stroke is essential. Recently, noninvasive brain stimulation, including transcranial direct current stimulation (tDCS), has been used for modifying cortical activities by enhancing ipsilesional cortical excitability, reducing contralesional cortical excitability, or both [5]. tDCS induces a subthreshold shift of resting membrane potentials toward hyperpolarization or depolarization [6]. Anodal-tDCS enhances long-lasting alterations in cortical excitability, whereas cathodal-tDCS reduces those alterations [7]. Regarding the effects of tDCS on motor function in patients with stroke, several studies have reported improvement in motor ability [8]. However, the improvement in motor disability as reported in these studies is limited, and tDCS in combination with motor training and not alone is recommended [5]. Several reports have shown the effectiveness of tDCS combined with other therapies [9]. Furthermore, peripheral neuromuscular electrical stimulation (PNES) has been reported to be effective when combined with tDCS and other therapies. Uy and Ridding [10] have suggested that combining peripheral nerve stimulation (PNS) with anodal-tDCS can prolong cortical excitability; therefore, combining tDCS and electrical stimulation may be a good method. Moreover, other studies have reported that tDCS combined with electrical stimulation, including neuromuscular electrical stimulation (NMES), is effective in improving affected upper extremity motor function [11].

Constraint-induced movement therapy (CIMT) is a promising motor training used in combination with tDCS. It improves upper extremity motor function in patients with chronic stroke [12]. Its mechanism for improving interhemispheric competition might be similar to that of tDCS [13]. Previous studies have reported that CIMT combined with anodal-tDCS was effective for treating upper extremity motor function in patients with stroke [14]. Additionally, Takebayashi et al. have suggested that CIMT combined with dual-hemisphere tDCS (dual-tDCS) and PNES provide meaningful upper extremity motor function improvements in patients with chronic stroke [15]. In their study, the anode was placed over the affected area of the primary motor cortex, whereas the cathode was placed over the contralateral primary motor cortex.

Previous studies have demonstrated the effects of CIMT combined with tDCS alone or tDCS and electrical stimulation on affected upper extremity motor function. As there are various stimulation sites, intensities, and durations in previous studies, there is insufficient evidence to generate recommendations about the treatment. Therefore, investigating the effects of CIMT combined with tDCS is necessary. We have been routinely performing CIMT in general practice on patients with chronic stroke. In some of them, to promote improvement in upper limb function, we have performed CIMT combined with tDCS and PNES. Therefore, we decided to retrospectively observe the effects of CIMT to date. This retrospective study aimed to determine whether CIMT combined with tDCS and PNES promotes upper extremity motor function improvement compared with CIMT alone.

## Materials and methods

Design

A retrospective design was used to compare the efficacy of CIMT combined with tDCS and PNES and CIMT alone for patients with chronic stroke for six months following ischemic or hemorrhagic stroke. This study was conducted following the Declaration of Helsinki and was approved in 2018 by the Hyogo Medical University Research Ethics Committee (approval number 3056).

Patients

This study included patients who underwent CIMT during outpatient occupational therapy at Hyogo College of Medicine Hospital between 2012 and 2018. Those who opted to undergo CIMT came to our hospital. 

The following were the inclusion criteria of CIMT: (1) age ≥ 20 years, (2) >6 months following ischemic or hemorrhagic stroke, (3) active extension of the wrist by >20° and extension of the metacarpophalangeal and interphalangeal joints of ≥2 fingers by >10°, and (4) safe walking. 

The following were the exclusion criteria: (1) did not complete the training, (2) did not complete all evaluations, (3) age < 20 years, and (4) injection of botulinum toxin within three months.

tDCS and PNES

A previous study reported that tDCS and PNES were performed before CIMT to prolong cortical excitability for a longer period [10]. The patients in the CIMT combined with tDCS and PNES group (experimental group) received anodal-tDCS and PNES before morning and afternoon CIMT. Anodal-tDCS was delivered using DC-STIMULATOR PLUS (NeuroConn GmbH, Ilmenau, Germany). To stimulate the primary motor cortex, the anode electrode was placed over the C3 or C4 according to the 10-20 system [16], whereas the cathode electrode was placed over the contralateral supraorbital area. A constant current of 1-mA intensity [7] was applied for 20 min [17]. PNES was performed immediately following anodal-tDCS using a TORIO stimulation system (Ito Co., Ltd., Tokyo, Japan). The two electrodes were placed on the extensor digitorum muscle. Trains of electrical stimulation (frequency = 25 Hz and pulse duration = 300 µs) were administered for 10 min. Stimulus intensity was set such that each patient reported mild paresthesia without pain and a minimal visible contraction was observed [10].

CIMT

Under the supervision of trained occupational therapists who were not blinded, CIMT was performed in each group for 10 consecutive weekdays. The evaluation took two hours and was performed in the morning of the first day and the afternoon of the last day. Therefore, the training performed on the first and last days lasted for three hours in the afternoon of the first day and two hours in the morning of the last day. In each group, five hours of CIMT were performed (two and three hours in the morning and afternoon, respectively) on the other eight days. Therefore, the total CIMT time was 45 hours.

The CIMT protocol involved the following three main elements: (1) repetitive task-oriented training of the affected arm; (2) the transfer package, designed to facilitate the transfer of therapeutic gains incurred in the clinical setting to the patient’s real-world activities; and (3) restraining of the unaffected arm to enforce the use of the affected arm [18].

In the task-oriented training, the duration of each task was approximately 10-15 min, and shaping and task practice were performed. Shaping is an approach that focuses on motor function. In this approach, patients perform tasks, including placing blocks to acquire the target motor function. The difficulty level of the task was progressively increased according to the condition of the patient’s upper limbs (e.g., the size and form of the object as well as the distance and direction to transfer the object). Moreover, the task practice leads to performing activities (e.g., writing and using scissors). The transfer package provided three main interventions for 30 min daily for the facilitation of the use of an affected arm in ADLs. First, the patient contracted with the therapist to intensively use the affected arm in ADLs. Second, keeping a home diary and reporting on the use of the affected arm were required. Lastly, the therapist discussed with the patient why the affected arm could not be used in ADLs and how to resolve the same.

Examination

The evaluation was performed in the morning of the first day and in the afternoon of the last day. Occupational therapists who were not blinded performed the evaluation. The Fugl-Meyer assessment (FMA) was performed for the primary outcome measure for the upper extremity [19]. It was used to evaluate the upper extremity measures on the assessment of impairments. To assess secondary outcome measures, the Action Research Arm Test (ARAT) and the Motor Activity Log (MAL) were used [20, 21]. ARAT was used for evaluating the assessment of limitations in activity, whereas MAL was used for evaluating how much (amount-of-use [AOU] scale) and how well (quality-of-movement [QOM] scale) the affected upper extremity was used in ADLs.

FMA for the upper extremity

The FMA for the upper extremity consists of 33 items that evaluate basic arm function, coordination, and speed [19]. The total score for the assessment ranges from 0 to 66 points on a three-point ordinal scale. The FMA has high reliability and validity in patients following stroke using the standardized manual [22].

Action Research Arm Test (ARAT)

The ARAT consists of 19 items that evaluate a patient’s ability to handle blocks and objects of different sizes [20]. It is subdivided into the following four categories: grasp, grip, pinch, and gross movement. The total score for the assessment ranges from 0 to 57 points on a four-point ordinal scale. The ARAT has high reliability and validity in patients following stroke using the standardized manual [22].

Motor Activity Log (MAL)

The MAL is a structured interview consisting of 14 specific ADLs. The questionnaire rates how often (AOU) and how well (QOM) ADL tasks are performed using the affected arm in the real-world setting by using a six-point scale ranging from 0 to 5 [21].

Statistical analysis

Statistical analyses were performed using SPS (version 22, IBM, Armonk, NY, USA) and R statistical package (version 3.4.0; R Foundation for Statistical Computing, Vienna, Austria). The chi-square test or Mann−Whitney U test was used to analyze between-group differences in baseline characteristics. The Wilcoxon signed-rank test was used to compare within-group differences to determine the effects before and after the intervention. The Mann-Whitney U test was used to compare between-group differences in motor-related outcomes and determine whether CIMT combined with tDCS and PNES promotes upper extremity motor function improvement compared with CIMT alone. A p-value of <0.05 was considered statistically significant.

## Results

**Figure 1:**
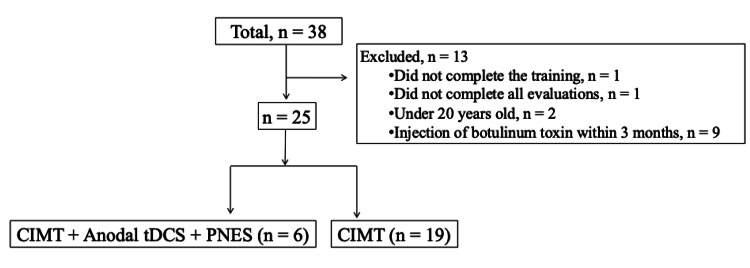
Figure 1. Flowchart of patient enrollment in the study CIMT, constraint-induced movement therapy; tDCS, transcranial direct current stimulation; PNES, peripheral neuromuscular electrical stimulation

Of 38 patients, 13 were excluded. A total of 25 patients were analyzed. The experimental and control groups consisted of 6 and 19 patients, respectively (Figure 1). Patients’ baseline characteristics in each group are presented in Table 1. No significant difference was observed between the two groups regarding baseline characteristics.

**Table 1:** Patients’ characteristics at baseline To analyze between-group differences in baseline characteristics, the chi-square test was used for categorical data, and the Mann-Whitney U test was used for continuous data. IQR, interquartile range; FMA, Fugl–Meyer assessment; ARAT, Action Research Arm Test; MAL, Motor Activity Log; AOUm amount-of-use; QOM, quality-of-movement

Characteristics	Experimental group (n = 6)		Control group (n = 19)	P-value
Median age (IQR)	55.5 (50.5−59.8)	53.0 (44.5−62.5)		0.98
Sex, male/female	3/3	13/6		0.41
Median months since stroke (IQR)	32.2 (22.5−50.3)	15.0 (10.0−37.5)		0.14
Hemiparesis, right/left	4/2	8/11		0.29
Hand dominance, right/left	5/1	19/0		0.07
Stroke type, hemorrhage/infarction	2/4	7/12		0.88
Median FMA for upper extremity(IQR)	46.5 (41.3−51.0)	48.0 (37.5−54.0)		0.78
Median ARAT (IQR)	38 (35.8−38.0)	39 (30.5−53.0)		0.51
Median MAL AOU (IQR)	1.48 (1.03−2.37)	1.57 (1.08−3.04)		0.93
Median MAL QOM (IQR)	1.37 (0.98−2.13)	1.64 (1.00−2.63)		0.78

Both groups showed significant improvement in all outcomes following CIMT (p < 0.05) (Table 2). The FMA showed a significant difference between the groups in the CIMT combined with anodal-tDCS and PNES group (p = 0.047) (Table 3).

**Table 2:** Within-group comparisons of outcomes Data are presented as medians (interquartile ranges). The Wilcoxon signed-rank test was used to compare the differences within groups in motor-related outcomes. FMA, Fugl–Meyer assessment; ARAT, Action Research Arm Test; MAL, Motor Activity Log; AOU, amount-of-use; QOM, quality-of-movement

Outcome	Experimental group (n = 6)			Control group (n = 19)		
	Baseline	Post treatment	P-value (r-value)	Baseline	Post treatment	P-value (r-value)
FMA	46.5 (41.3−51.0)	53.5 (51.3−56.5)	0.027 (0.9)	48.0 (37.5−54.0)	54.0 (43.0−62.0)	<0.001 (0.77)
Improvement	7 (5.5−10)			4 (1.5−6.5)		
ARAT	38.0 (35.8−38.0)	40.5 (38.3−42.0)	0.039 (0.84)	39.0 (30.5−53.0)	47.0 (33.5−55.0)	0.001 (0.76)
Improvement	4 (4−4.8)			3 (1−7.5)		
MAL AOU	1.48 (1.03−2.37)	3.09 (2.03−3.27)	0.028 (0.9)	1.57 (1.08−3.04)	2.96 (1.79−3.87)	<0.001 (0.88)
Improvement	0.6 (0.4−1.5)			0.6 (0.4−1.1)		
MAL QOM	1.37 (0.98−2.13)	2.90 (1.94−3.18)	0.028 (0.9)	1.64 (1.00−2.63)	2.70 (1.69−3.36)	<0.001 (0.85)
Improvement	0.8 (0.6−1.5)			0.7 (0.5−1)		

**Table 3:** Outcome between-group comparisons The Mann–Whitney U test was used to compare between-group differences in motor-related outcomes. CI, confidence interval; FMA, Fugl–Meyer assessment; ARAT, Action Research Arm Test; MAL, Motor Activity Log; AOU, amount-of-use; QOM, quality of movement

Outcome	Difference	95% CI	P-value	r-value
FMA	3.00	3.6−6.6	0.047	0.398
ARAT	1.50	2.3−10.1	0.201	0.256
MAL AOU	−0.02	0.5−1.0	0.799	0.051
MAL QOM	0.12	0.6−1.0	0.524	0.128

## Discussion

In this study, both groups showed significant improvement in all outcomes following CIMT. Furthermore, our main finding was that a significant difference was noted in the FMA improvement with CIMT plus preconditioning stimulations compared with CIMT alone. Our findings indicate that preconditioning with anodal-tDCS and PNES may promote upper extremity function improvement in CIMT.

Several previous studies have reported that CIMT combined with anodal-tDCS has a significant difference in upper extremity motor function, including the FMA and Wolf Motor Function Test in patients with chronic stroke, compared with CIMT alone [14]. In studies regarding combined CIMT with anodal-tDCS, there were no reports (such as the MAL) that evaluated using the affected arm in the real-world setting. There is another study regarding dual-tDCS, which simultaneously uses both anodal- and cathodal-tDCS [23]. In the present study, although the MAL was used for evaluation, no significant difference was noted regarding using the MAL compared with CIMT alone. However, Takebayashi et al. [15] have reported interesting results of CIMT combined with dual-tDCS and PNES. They have mentioned that significant differences were observed in both the FMA upper extremity and MAL AOU scores. In our study, CIMT combined with anodal-tDCS and PNES showed a significant difference only in the FMA. Therefore, using dual-tDCS might be promising for improving upper limb function. However, there has been no report examining the difference in the effects of CIMT depending on whether anodal-tDCS or dual-tDCS was used. To verify effective ways to use tDCS, future studies should directly compare combining anodal-tDCS and combining dual-tDCS.

The minimum clinically important difference (MCID) in patients with chronic stroke has been demonstrated as an improvement of >4.25 points in the FMA upper extremity score [24], >5.7 points in the ARAT score [25], and >0.5 points in the MAL AOU score [26]. Regarding the MAL QOM, the MCID has been shown as above 1.0 point in patients with acute stroke [27]. In our study, only the experimental group exhibited improvements above the MCID on the FMA upper extremity score. Both experimental and control groups exhibited improvements above the MCID on the MAL AOU score. Furthermore, a significant difference was observed in the FMA improvement with CIMT plus preconditioning stimulations compared with CIMT alone. These results suggest that CIMT combined with anodal-tDCS and PNES may promote affected arm function improvement, such as in the FMA upper extremity score.

Interhemispheric suppression relationship normalization by manipulating the threshold value of spontaneous firing of single neurons in direct current stimulation is one of the possible mechanisms that are considered regarding the superiority of training using these tDCS in combination [4, 23]. Furthermore, Uy and Ridding [10] have suggested that long-term excitability of the cerebral cortex by tDCS lasts longer than stimulation by tDCS alone when combined with PNES following tDCS stimulation. Therefore, the experimental group might have trained the upper extremity with more enhancing cortical excitability versus no preconditioning.

In our study, the ARAT and MAL scores did not show a significant difference. Regarding the ARAT score, the high score of the participants in our study might have been affected. Although the maximum total score of the ARAT is 57 points, the baseline total scores of the ARAT in our study were the high scores, which were 38 (35.8-38.0) and 39 (30.5-53.0) points in the intervention and control groups, respectively. The ARAT has ceiling effects [28, 29] because it only employs the ordinal four-point scale system. Since our other studies have suggested that the performance timescale on the ARAT score scale partially solves the concern of the ceiling effect, we measured the performance timescale [30]. Regarding the no significant difference observed in the MAL score, this might have been affected by the transfer package. In CIMT, the therapeutic gains made in the clinical setting are incorporated into the patient’s real-world activities through the transfer package [18]. In other words, the task-oriented training might promote the FMA upper extremity score improvement as upper limb function, and the transfer package might promote the MAL score improvement as upper limb use. Although Uy and Ridding [10] have suggested that combining PNS with tDCS promotes cerebral excitability for 30 min following stimulation, the effect at 30 min following stimulation is unclear. Only task-oriented training might have been affected following preconditioning stimulations because tDCS and PNES were used in our study shortly before the task-oriented training. Therefore, the effects of tDCS and PNES may have influenced improvements at the impairment level rather than at the activity level, with only the FMA showing a significant difference.

This study had some limitations. First, this study was designed as a single-center retrospective study. Second, because this study was designed as a retrospective analysis, it did not employ a formal sampling procedure or methods for distributing patients across the two groups. As a result, the sample size was small, and there was a bias in the number in each group. Therefore, the results of this study may not be generalizable. Lastly, since the design of this study compares preconditioning CIMT, including tDCS and PNES and CIMT alone, examining the effects of using tDCS and PNS alone was not possible. To generate evidence for CIMT combined with preconditioning stimulations, additional studies of adequate size and rigorous design are needed, such as those employing a prospective design and a clear procedure for selecting appropriate subjects and sample sizes.

## Conclusions

This study demonstrates that CIMT combined with anodal-tDCS and PNES improves upper extremity motor function in patients with chronic stroke compared with CIMT alone. Preconditioning interventions, such as tDCS and PNES, may be good methods for further enhancing the effectiveness of CIMT.
